# Anatomical variations of the pronator teres muscle in a Central European population and its clinical significance

**DOI:** 10.1007/s12565-017-0413-y

**Published:** 2017-08-28

**Authors:** Łukasz Olewnik, Michał Podgórski, Michał Polguj, Grzegorz Wysiadecki, Mirosław Topol

**Affiliations:** 1 Department of Normal and Clinical Anatomy, Interfaculty Chair of Anatomy and Histology, Medical University of Lodz, Lodz, Poland; 2 Department of Angiology, Interfaculty Chair of Anatomy and Histology, Medical University of Lodz, Lodz, Poland

**Keywords:** Anatomical variations, Forearm, Innervation, Median nerve, Pronator teres muscle, Pronator teres syndrome

## Abstract

The pronator teres (PT) muscle is a forearm flexor with radial and ulnar heads. It is innervated by the median nerve (MN), which passes between these heads. Nerve entrapment, known as “PT syndrome”, may occur in this passage. Anatomical variations in this region may be potential risk factors of this pathology. Therefore, the aim of the study was to determine the relationship between morphologic variations of the PT and the MN. In 50 isolated, formalin-fixed upper limbs, the cubital region and the forearm were dissected. The following measurements were taken: origin of the PT muscle heads, the length of these heads, the length of the forearm, diameter of the MN and the number of its muscular branches to the pronator teres muscle. The forearms with the humeral head originating from the medial humeral epicondyle and medial intermuscular septum (72%) were significantly shorter (*p* = 0.0088) than those where the humeral head originated only from the medial humeral epicondyle. Moreover, in these specimens, the MN was significantly thinner (*p* = 0.003). The ulnar head was present in 43 limbs (86%). The MN passed between the heads of the PT muscle (74%) or under the muscle (26%). In the majority of cases, it provided two motor branches (66%). There is an association between the morphologic variation of the PT muscle heads and the course and branching pattern of the MN. Both are related to differences in forearm length. This may have an impact on the risk of PT syndrome and the performance of MN electrostimulation.

## Introduction

The pronator teres (PT) is a fusiform muscle located mostly laterally in a superficial layer of the anterior forearm muscles (Moore and Dalley 2010; Bergman et al. 2015). It has two heads: humeral and ulnar. The humeral head attaches proximally to the medial intermuscular septum of the arm and to the medial epicondyle of the humerus. The ulnar head originates from the coronoid process. Both heads run diagonally downwards and merge to form a common flexor tendon, which inserts at the middle of the lateral surface of the radius (Moore and Dalley 2010; Bergman et al. 2015). Typically, the radial artery passes anterior to the PT tendon. However, in rare cases, the radial artery may run posterior to the PT (Wysiadecki et al. 2017).

The PT is innervated by the median nerve (MN) that passes between the heads of the PT and then travels inferior to the flexor digitorum superficialis (Moore and Dalley 2010; Bergman et al. 2015). In the cubital region, this narrow passage through the PT may be the location for MN compression or entrapment, known as pronator syndrome. The issue is still clinically important. Although magnetic resonance imaging and ultrasonography have facilitated the diagnosis of median nerve entrapment (Chen et al. 2011; Asheghan et al. 2016; Zamborsky et al. 2017), pronator syndrome may be easily overlooked and mistaken for the much more frequent carpal tunnel syndrome (Lee and LaStayo 2004; Bilecenoglu et al. 2005; Dang and Rodner 2009; Afshar 2015; Vymazalová et al. 2015). Pronator syndrome may manifest with pain in the PT region as well as paresthesia and dysthesia or paralysis in the median nerve innervation zone (Lee and LaStayo 2004; Bilecenoglu et al. 2005; Andreisek et al. 2006; Dang and Rodner 2009; Miller and Reinus 2010; Vymazalová et al. 2015).

Entrapment syndrome of the MN in the proximal part of the forearm may be caused by, among other factors, morphologic variability of the PT muscle (Bilecenoglu et al. 2005; Vymazalová et al. 2015). Pronator teres syndrome is a complex group of neuropathies associated with the compression of the median nerve by the PT muscle or fibrous band extending from the surface of the humeral head of the PT. Compression of the MN in the proximal part of the forearm may be caused by the following anatomical structures: the PT muscle having two heads of origin, the presence of a proximal arch to the flexor digitorum superficialis muscle, lacertus fibrosus in the antecubital fossa or an anomalous Struther ligament (Bilecenoglu et al. 2005; Camerlinck et al. 2010; Afshar 2015; Vymazalová et al. 2015). One possible form of compression of the MN in the proximal part of the forearm is caused by variation in the humeral and ulnar heads of the PT (Bilecenoglu et al. 2005; Andreisek et al. 2006; Xing and Tang 2014; Asheghan et al. 2016).

The aim of this study is to determine the correlation between the morphology of the PT and the course and number of muscular branches of the MN.

## Materials and methods

**Fig. 1:**
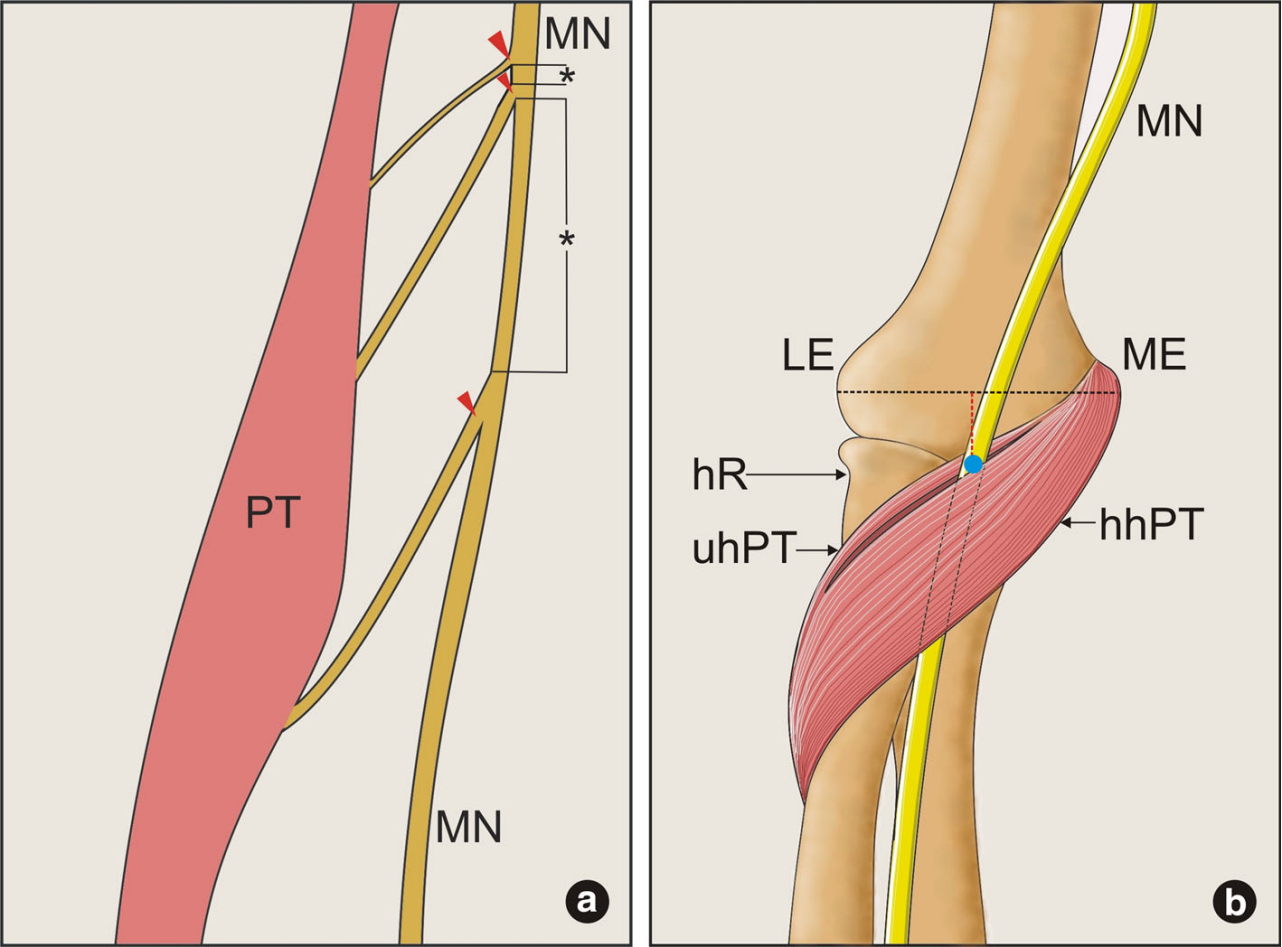
**a** Schematic drawing of the median nerve measurements. *Asterix* (*) show distances between the branches of the median nerve; *red arrowheads* show the location of the measurement of the nerve diameter. *PT* pronator teres muscle, *MN* median nerve. **b** Schematic drawing of an intercondylar line (*black dotted line*) and location of the median nerve passage through the pronator teres muscle. The *blue circle* shows the location where the diameter of the median nerve was evaluated. The distance between this point and the intercondylar *line* was measured as indicated by *red dotted line*. MN median nerve, *LE* lateral epicondyle, *ME* medial epicondyle, *hR* head of radius, *uhPT* ulnar head of the pronator teres muscle, *hhPT* humeral head of the pronator teres muscle

Fifty formalin-fixed, randomly selected, isolated upper limbs (26 left and 24 right) were obtained from adult cadavers. Permission for the study was given by the Local Bioethics Commission (agreement no. RNN/44/16/KE). A dissection of the cubital region and the proximal part of the forearm was performed by using traditional techniques (Jamieson and Anson 1952; Olewnik et al. 2017a, b; Wysiadecki et al. 2016). After the median nerve had been exposed, the dissection of its muscular branches was performed. The diameter of both the nerve and its branches was measured (Fig. 1a). The nerve was measured just above the first muscular branch of the PT (Fig. 1a). The level of branching was assessed by measuring the distance from the intercondylar line to the point of origin of the muscular branch (Fig. 1b). The level of passage of the MN through the PT muscle was characterized in the same manner (Fig. 1b). The lengths of the humeral and ulnar heads of the PT muscle were then measured by taking the distance between the furthest points of their origins and common insertion. All measurements were taken twice with an accuracy of up to 0.1 mm using an electronic digital caliper (Mitutoyo Corp., Kawasaki-shi, Kanagawa, Japan). The length of the whole forearm was also measured as the distance between the olecranon and the styloid processes of the ulna.

Statistical analysis was performed with Statistica software. A *p* -value < 0.05 was considered significant. Data were presented as mean and standard deviation unless otherwise stated. Nominal variables (e.g., type of MN course and number of MN muscular branches) were compared based on contingency tables and the Chi^2^ test, with appropriate corrections. The distribution of continuous data was evaluated with the Shapiro–Wilk test; non-parametric tests were then applied to evaluate non-normal data. The Mann–Whitney test was used to compare measurements regarding the number of PT heads or their origin between two groups. The Kruskal–Willis ANOVA with dedicated post hoc test was used to compare muscle/forearm dimensions according to the number of MN muscular branches. Correlations were assessed with the Spearman’s rank correlation coefficient.

## Results

The results section is divided into two parts. The first part concerns the morphology of the PT and its association with the course of the MN, while the second describes variations in the number of muscular branches supplying the PT. In all cases, the radial artery ran in front of the PT tendon.

### Variation in PT morphology and its association with the morphology of the MN and forearm length

**Fig. 2:**
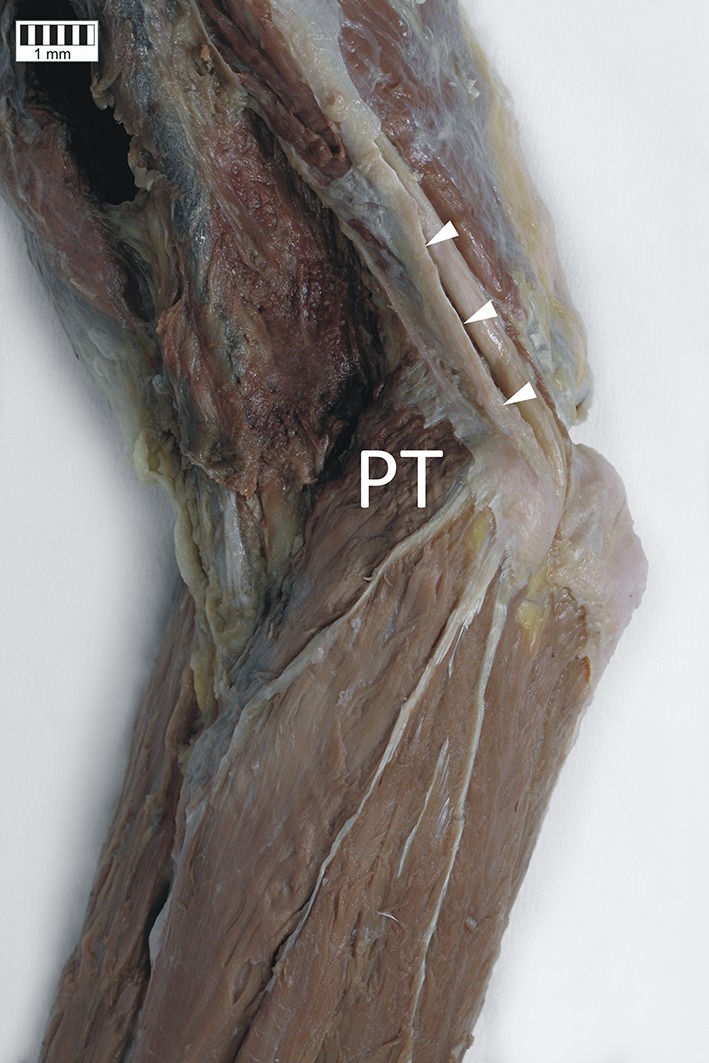
Origin of the pronator teres muscle. *PT* pronator teres muscle, *white arrowheads* indicate the medial intermuscular septum

Two types of PT origin were observed. In 36 cases (72%), the humeral head of the PT originated from the medial humeral epicondyle and medial intermuscular septum (Fig. 2), while in the remaining 14 specimens (28%), its origin was only on the medial humeral epicondyle. The mean length of the forearm when the PT originated both from the medial humeral epicondyle and medial intermuscular septum (288 ± 20 mm) was significantly shorter than in cases when the PT origin was only on the medial humeral epicondyle (306 mm ± 12) (*p* = 0.0088). Furthermore, in the former group, the MN was significantly thinner (5.6 ± 0.6 mm) than in specimens with the PT origin located only on the medial humeral epicondyle (6.3 ± 0.7 mm) (*p* = 0.003). The ulnar head of the PT was present in 43 limbs (86%), and in all cases it originated from the coronoid process of the ulna. Forearms where the ulnar head was absent were significantly longer (310 ± 10 mm vs. 294 ± 20 mm; *p* = 0.0397) and had significantly wider nerves (6.2 ± 0.2 mm vs. 5.9 ± 0.7) than those where it was present.

No gross variation was observed for the location of the insertion: in all specimens, the PT attached to the lateral surface of the radius.

### Types of the MN course in relation to the PT

The median nerve was observed to follow three course variants in relation to the PT:

1. The MN passed between the two heads of the PT muscle: 37 cases (74%) (Fig. 3a).

**Fig. 3:**
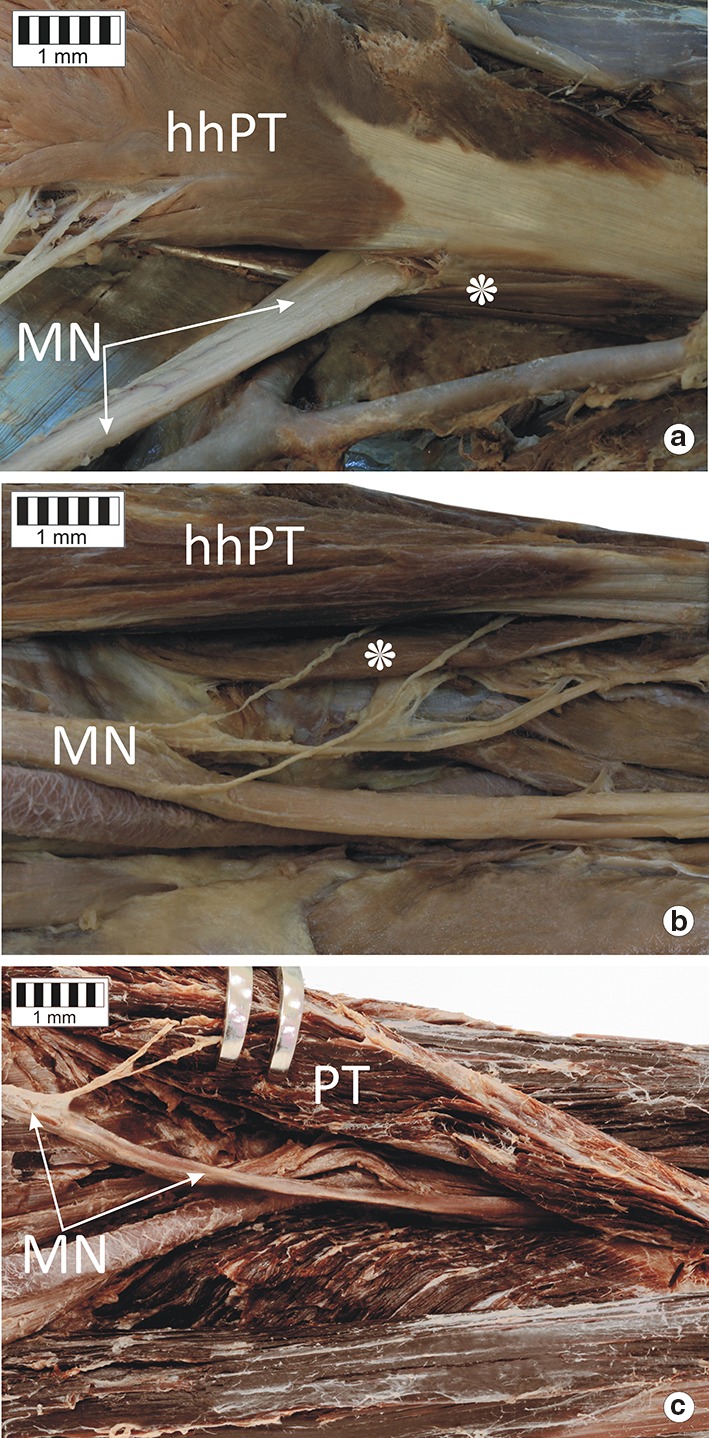
Types of course of the median nerve. **a** Type I of the median nerve course. **b** Type II of the median nerve course. **c** Type III of the median nerve course. *MN* median nerve, *hhPT* humeral head of the pronator teres muscle, *PT* pronator teres muscle, *asterisk* ulnar head of the pronator teres muscle
2. The MN passed under two heads of the PT: 6 cases (12%) (Fig. 3b).
3. The PT possessed only a humeral head, and the MN passed deep to it: seven cases (14%) (Fig. 3c).

There was no significant difference in any of the analyzed measurements according to the type of MN course (Table 1). However, significant positive correlations were observed between the MN diameter and the lengths of the forearm (*R* ^2^ = 0.60, *p* = 0.0001) and the radial head of the PT muscle (*R* ^2^ = 0.59, *p* = 0.0001), but not with the length of the ulnar head (*R* ^2^ = 0.24, *p* = 0.1291). On the other hand, a significant negative correlation was found only between the distance from the intercondylar line to the passage of the MN and the length of the ulnar head (*R* ^2^ = −0.47, *p* = 0.0014; *R* ^2^ = 0.27, *p* = 0.0625 for the forearm and *R* ^2^ = −0.01, *p* = 0.9823 for the length of the radial head).

**Table 1:** Comparison of performed measurements according to MN morphology

Parameter		Length (mm)			MN diameter (mm)	Distance MN − intercondylar line (mm)
		Forearm	PT radial head	PT ulnar head		
MN course*	Between	293 (20)	135 (8)	94 (7)	5.9 (0.8)	53 (7)
	Behind	303 (16)	140 (4.8)	100 (3)	6 (0.4)	53 (8)
*p* value		0.1809	0.0541	0.1147	0.2000	0.7907
Number of MN branches†	1	310 (9)	136 (5)	88 (4)	6.1 (0.2)	60 (4)
	2	299 (15)	140 (4)	99 (5)	6.1 (0.7)	51 (6)
	3	266 (6)	123 (3)	90 (3)	4.9 (0.1)	50 (7)
*p* value		0.0001	0.0001	0.0001	0.0001	0.0057

Applied test

^a^ The Mann–Whitney test

^b^The Kruskal–Willis ANOVA

### Muscular branches of the MN to the PT

**Fig. 4:**
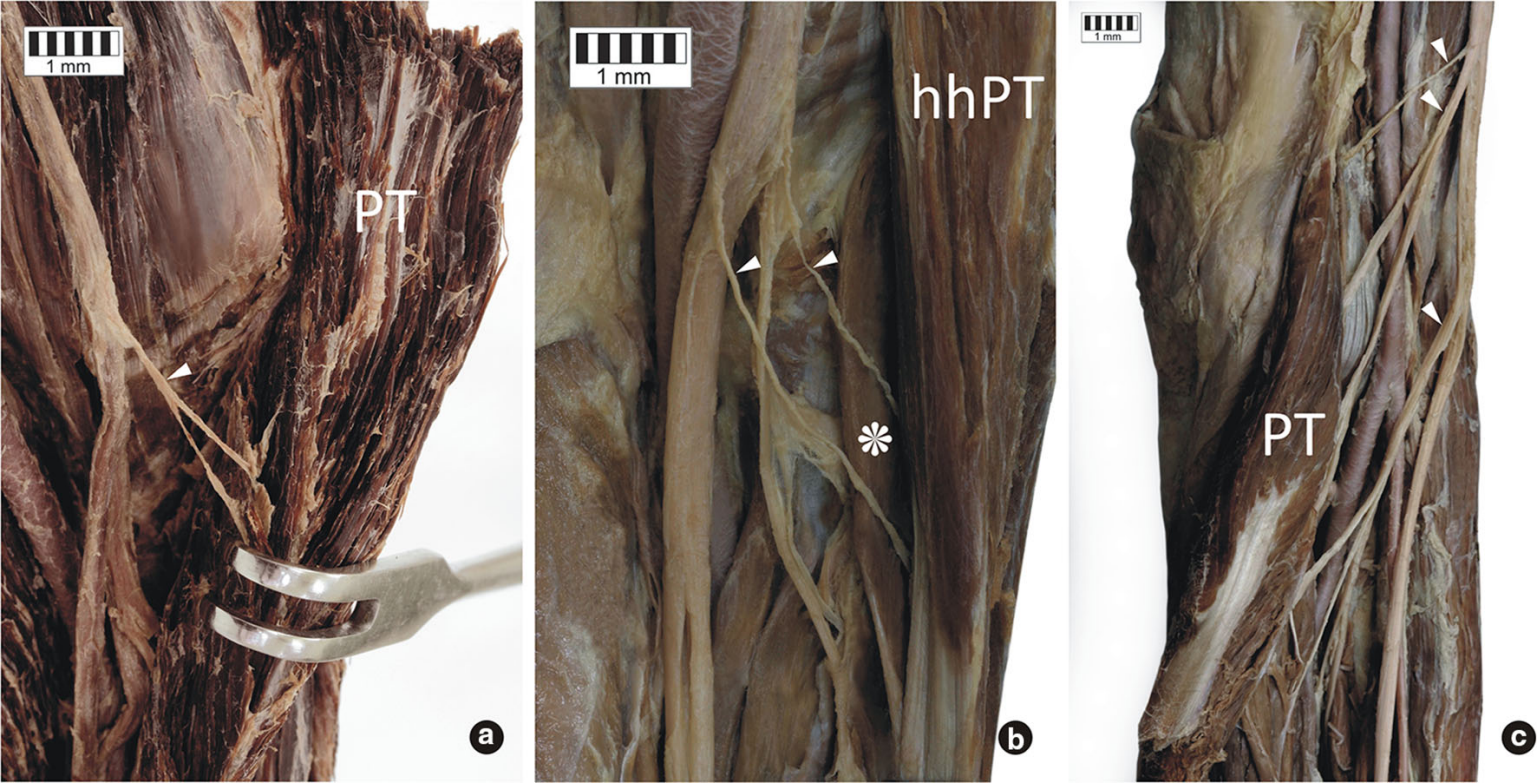
Variants of the innervation of the pronator teres muscle. **a** Variant C of the innervation of the pronator teres muscle. **b** Variant A of the innervation of the pronator teres muscle **c** Variant B of the innervation of the pronator teres muscle. *PT* pronator teres muscle, *hhPT* humeral head of the pronator teres muscle, *asterisk* ulnar head of the pronator teres muscle, *white arrowheads* indicate the muscular branches of the median nerve

A few variants were found in the number of muscular branches to the PT arising from the MN. If both heads of the PT were present, there were three types of PT innervation pattern: type I—with one muscular branch arising from the main trunk of the MN, this type occurred in seven cases (14%)—(Fig. 4a); type II—with two muscular branches arising from the main trunk of the MN, this type occurred in 26 cases (52%)—(Fig. 4b); and type III with three muscular branches arising from the main trunk of the MN, this type occurred in 10 cases (20%)—(Fig. 4c).

In all the cases where only the humeral head of the PT was present (7 cases, 14%), two muscular branches arising from the main trunk of the MN were observed. Selected parameters were correlated with the number of muscular branches of the MN (Table 1). When three muscular branches were present, the lengths of the forearm and radial heads of the PT were significantly shorter, and the MN nerve was significantly narrower, than in the two other types. However, when two muscular branches were present, the ulnar head of the PT was significantly longer than in the two other types. The distance from the intercondylar line to the passage of the MN through the PT muscle was significantly longer in the type with one muscular branch than in the two other types.

## Discussion

The present study describes the morphologic variations of the origins of the PT and their association with the course and branching pattern of the MN. Entrapment syndromes of the MN are still an important clinical problem. Thus, our research provides a valuable data and knowledge concerning morphologic variations as a potential risk factor of MN entrapment neuropathy.

Entrapment neuropathies within the upper limb can be diagnosed with both ultrasound and magnetic resonance imaging. However, due to the lower cost and dynamic character of the examination, ultrasound seems to present important advantages (Chen et al. 2011; Asheghan et al. 2016; Zamborsky et al. 2017). In a group of patients suspected to have MN entrapment, (Asheghan et al. 2016) performed a dynamic ultrasound evaluation of the position of the MN between the humeral and ulnar heads of the PT during pronation of the forearm, measuring the cross section of the nerve above, below and at the site of passage through the PT. A similar study was conducted by Chen et al. (2011). Both authors demonstrated that the cross-sectional area of the MN positively correlated with nerve conduction failure, as well as the severity and duration of symptoms (Chen et al. 2011; Asheghan et al. 2016). Summing up, the criterion of MN entrapment should include any noticeable reduction in cross-sectional diameter and reduced mobility of the nerve during the flexion, supination and pronation of the hand (Chen et al. 2011; Asheghan et al. 2016).

Concerning the ethology, compression of the MN in the region of the elbow joint has been associated with the morphology of the PT or with the presence of Struther’s ligament or a supracondylar process of the humerus bone (Bilecenoglu et al. 2005; Camerlinck et al. 2010; Vymazalová et al. 2015). Due to the rare occurrence of Struther’s ligament and the supracondylar process, or ignorance of the possible course of the MN through the PT, the condition may be misdiagnosed as a similar condition, such as carpal tunnel syndrome, and result in inappropriate treatment being given (Lee and LaStayo 2004; Vymazalová et al. 2015). The absence of the ulnar head may reduce the risk of entrapment of the MN.

In our study, the distribution of the origin types of the PT humeral head was found to be comparable to those identified in former studies. A previous study by Vymazalová et al. (2015) found the PT to be attached at both the medial humeral epicondyle and the medial intermuscular septum in 70.6% of cases compared to 72% of cases in the present study. The same authors reported a single origin of the humeral head from the medial humeral epicondyle in 29.4% of cases (Vymazalová et al. 2015), which again is comparable with the 28% identified in the present study. In contrast to previous studies (Nebot-Cegarra et al. 1991; Vymazalová et al. 2015), no examples of PTs with double humeral heads were found.

In the present study, the ulnar head of the PT always originated from the coronoid process of the ulna, although a previous study found it to attach to the trochlea of the humerus in 3.1% of examined cases (Vymazalová et al. 2015). Nebot-Cegarra et al. (1991) and Vymazalová et al. (2015) identified three types of ulnar head: muscular, tendinous and mixed. The ulnar head of the PT was characterized by high morphologic variability, with both the muscular (Vymazalová et al. 2015) and tendinous forms (Nebot-Cegarra et al. 1991) being found to predominate; however, only the muscular type was found in the present study. From the clinical point of view, the tendinous ulnar head is important because it carries significantly more tension than the muscular head, which can increase the degree of compression to the median nerve (Nebot-Cegarra et al. 1991; Vymazalová et al. 2015). The frequency of the absence of the PT humeral head varies between studies by different authors. Nebot-Cegarra et al. (1991) found the ulnar head to be absent in 22% of limbs, Vymazalová et al. (2015) in 4.4% of cases, while Bilecenoglu et al. (2005) found it to be absent in one out of 30 cases. In the present study, the ulnar head was absent in 14% of specimens.

The course of the MN in relation to the PT is clinically important because of the difficulty in diagnosing PT syndrome. Although neuropathy of the median nerve can vary significantly depending on the compression site, it also can be very similar and unambiguous (Lee et al. 2014). Many previous publications reported that PT syndrome had been mistakenly diagnosed as carpal tunnel syndrome or a similar pathology (Jabaley et al. 1980; Olehnik et al. 1994; Bridgeman et al. 2007; Lee et al. 2014). Neurologic tests are not always reliable; for example, the PT syndrome test and the Phalen test may give positive results in the case of both PT syndrome and carpal tunnel syndrome (Buchthal et al. 1974; Lee et al. 2014).

The course of the MN in relation to PT depends on the morphology of the muscle and its adjacent anatomical structures (Table 2) (Jamieson and Anson 1952; Mori 1964; Nebot-Cegarra et al. 1991; Vymazalová et al. 2015). Similarly to earlier reports (Jamieson and Anson 1952; Mori 1964; Nebot-Cegarra et al. 1991; Vymazalová et al. 2015), the most common MN course type observed in the present study was between the two heads of the PT (74% of cases). In our study, the MN ran beneath the humeral head (absent ulnar head) in 14% of cases. This course was also noted in the literature (Jamieson and Anson 1952; Mori 1964; Nebot-Cegarra et al. 1991; Vymazalová et al. 2015) and was classified as being the second most common course by both Jamieson and Anson (1952) and Nebot-Cegarra et al. (1991), similarly to our study. The least common of the three identified types, where the MN travels beneath the ulnar and humeral heads of the PT, was recognized in 12% of limbs, which was a higher frequency than seen in other studies. In contrast to former reports, the following types of PM morphology and MN course were not observed: a double humeral head with the MN running between them, passage of the MN through the ulnar head and passage of the MN between the humeral and ulnar heads of the PT alongside the ulnar artery.

**Table 2:** Comparison of the course of the median nerve in relation to the pronator teres muscle

Course of median nerve	Mori	Nebot-Cegarra et al.	Jamieson and Anson	Vymazalová et al.	Olewnik et al.
Between both heads	95%	75%	83.3%	85.3%	74%
Beneath both heads	0.25%	–	6%	2.9%	12%
Beneath HH	–	21.6%	8.7%	4.4%	14%
Through UH	–	3.4%	–	5.9%	–
Duplicate HH, through HH	0.25%	1.7%	2%	–	–
Together with UA	–	–	–	1.5%	–

*HH* humeral head, *UH* ulnar head, *UA* ulnar artery

Knowledge of the number of nerve branches is not only of value for the anatomist, physiotherapist and physiologist, but also for the clinician by allowing the appropriate type of electrostimulation (Safwat and Abdel-Meguid 2007; Wu et al. 2002). Electrical stimulation has gained popularity in the rehabilitation process in recent years. Functional electrical stimulation is used successfully to restore the motor function of paralyzed upper limbs or diseases of the upper motor neurons (Lau et al. 1995; Naito et al. 1991; Reilly and Schieber 2003; Safwat and Abdel-Meguid 2007). Planning electrical stimulation treatments is associated with knowledge of the muscular branches off the main nervous trunk.

Chantelot et al. (1999) differentiated a few types of MN muscular branches to the PT. They reported classical PT innervation, characterized by the presence of both the superior and inferior muscular branches, in only 26% of cases (Chantelot et al. 1999), while Safwat and Abdel-Meguid (2007) reported the presence of this type of PT innervation in all of 23 examined limbs (Table 3). Such ‘classical type’ of innervation was the most common variant identified in the present study, occurring in 26 cases (52%). Otherwise, the presence of a single muscular branch to the PT was reported in 56% cases by Chantelot et al. (1999) but in only 14% of cases in the present study, while the presence of three muscular branches to the PT was identified in one case by Chantelot et al. (1999) but in 20% of limbs in the present study. Accurate knowledge of the morphology of the PT muscle, its relationship with the median nerve and its innervation are important from the clinical point of view.

**Table 3:** Comparison of variants of innervation of the pronator teres muscle

Variant of innervation	Chantelot et al. (%)	Safwat et al.	Olewnik et al.
One muscular branch	56	–	14%
Two muscular branch	26	100%	52%
Three muscular branch	2	–	20%
Other	16	–	–

## Conclusions

An understanding of the different MN course types and variation in the insertions of the PT heads is essential in diagnosing patients with MN neuropathies. In the majority of cases, the median nerve passes between both heads of the pronator teres muscle. The knowledge that the cubital region is a potential site of proximal MN entrapment should be taken into account to better differentiate PT syndrome from carpal tunnel syndrome. Most often, the main trunk of the MN gave off two muscular branches to the PT. The knowledge of the number and location of muscular branches of the nerve may also be essential for the proper commencement of the electrostimulation process.

## References

[CR1] Afshar A (2015). Pronator syndrome due to Schwannoma. J Hand Microsurg.

[CR2] Andreisek G, Crook DW, Burg D, Marincek B, Weishaupt D (2006). Peripheral neuropathies of the median, radial, and ulnar nerves: MR imaging features. RadioGraphics.

[CR3] Asheghan M, Hollisaz MT, Aghdam AS, Khatibiaghda A (2016). The prevalence of pronator teres among patients with carpal tunnel syndrome: Cross-sectional study. Int J Biomed Sci.

[CR4] Bergman R, Afifi A, Miyauchi R (2015) Illustrated encyclopedia of human anatomic variation: opus I: muscular system: alphabetical listing of muscles, Pronator teres muscle. http://www.anatomyatlases.org/AnatomicVariants/AnatomyHP.shtml

[CR5] Bilecenoglu B, Uz A, Karalezli N (2005). Possible anatomic structures causing entrapment neuropathies of the median nerve: an anatomic study. Acta Orthop Belg.

[CR6] Bridgeman C, Naidu S, Kothari MJ (2007). Clinical and electrophysiological presentation of pronator syndrome. Electromyogr Clin Neurophysiol.

[CR7] Buchthal F, Rosenfalck A, Trojaborg W (1974). Electrophysiological findings in entrapment of the median nerve at wrist and elbow. J Neurol Neurosurg Psychiatry.

[CR8] Camerlinck M, Vanhoenacker FM, Kiekens G (2010). Ultrasound demonstration of Struthers’ ligament. J Clin Ultrasound.

[CR9] Chantelot C, Feugas C, Guillem P, Chapnikoff D, Rémy F, Fontaine C (1999). Innervation of the medial epicondylar muscles: an anatomic study in 50 cases. Surg Radiol Anat.

[CR10] Chen SF, Lu CH, Huang CR, Chuang YC, Tsai NW, Chang CC, Chang WN (2011). Ultrasonographic median nerve cross-section areas measured by 8-point “inching test” for idiopathic carpal tunnel syndrome: a correlation of nerve conduction study severity and duration of clinical symptoms. BMC Med Imaging.

[CR11] Dang AC, Rodner CM (2009). Unusual compression neuropathies of the forearm, part II: median nerve. J Hand Surg Am.

[CR12] Jabaley ME, Wallace WH, Heckler FR (1980). Internal topography of major nerves of the forearm and hand: a current view. J Hand Surg Am.

[CR13] Jamieson R, Anson B (1952). The relation of the median nerve to the heads of origin of the pronator teres muscle, a study of 300 specimens. Q Bull Northwest Univ Med Sch.

[CR14] Lau HK, Liu J, Pereira BP, Kumar VP, Pho RW (1995). Fatigue reduction by sequential stimulation of multiple motor points in a muscle. Clin Orthop Relat Res.

[CR15] Lee HJ, Kim I, Hong JT, Kim MS (2014). Early surgical treatment of pronator teres syndrome. J Korean Neurosurg Soc.

[CR16] Lee MJ, LaStayo PC (2004). Pronator syndrome and other nerve compressions that mimic carpal tunnel syndrome. J Orthop Sports Phys Ther.

[CR17] Miller TT, Reinus WR (2010). Nerve entrapment syndromes of the elbow, forearm, and wrist. Am J Roentgenol.

[CR18] Moore KL, Dalley AF, Agur MRA (2010). Clinically oriented anatomy.

[CR19] Mori M (1964). Statistics on the musculature of the Japanese. Okajimas Folia Anat Jpn.

[CR20] Naito A, Shimizu Y, Handa Y, Ichie M, Hoshimiya N (1991). Functional anatomical studies of the elbow movements. I. Electromyographic (EMG) analysis. Okajimas Folia Anat Jpn.

[CR21] Nebot-Cegarra J, Perez-Berruezo J, Reina de la Torre F (1991–1992) Variations of the pronator teres muscle: predispositional role to median nerve entrapment. Arch Anat Histol Embryol 74:35–45 1366347

[CR22] Olehnik WK, Manske PR, Szerzinski J (1994). Median nerve compression in the proximal forearm. J Hand Surg Am.

[CR23] Olewnik Ł, Wysiadecki G, Polguj M, Topol M (2017). Anatomic study suggests that the morphology of the plantaris tendon may be related to Achilles tendonitis. Surg Radiol Anat.

[CR24] Olewnik Ł, Wysiadecki G, Polguj M, Topol M (2017). The report on the co-occurrence of two different rare anatomic variations of the plantaris muscle tendon on both sides of an individual. Folia Morphol.

[CR25] Reilly KT, Schieber MH (2003). Incomplete functional subdivision of the human multitendoned finger muscle flexor digitorum profundus: an electromyographic study. J Neurophysiol.

[CR26] Safwat MD, Abdel-Meguid EM (2007). Distribution of terminal nerve entry points to the flexor and extensor groups of forearm muscles: an anatomical study. Folia Morphol.

[CR27] Vymazalová K, Vargová L, Joukal M (2015). Variability of the pronator teres muscle and its clinical significance. Rom J Morphol Embryol.

[CR28] Wu L, Goto Y, Taniwaki T, Kinukawa N, Tobimatsu S (2002). Different patterns of excitation and inhibition of the small hand and forearm muscles from magnetic brain stimulation in humans. Clin Neurophysiol.

[CR29] Wysiadecki G, Polguj M, Topol M (2016). Persistent jugulocephalic vein: case report including commentaries on distribution of valves, blood flow direction and embryology. Folia Morphol.

[CR32] Wysiadecki G, Polguj M, Haładaj R, Topol M (2017). Low origin of the radial artery: a case including a review of literature and proposal of an ambryological explanation. Anat Sci Int.

[CR30] Xing SG, Tang JB (2014). Entrapment neuropathy of the wrist, forearm, and elbow. Clin Plast Surg.

[CR31] Zamborsky R, Kokavec M, Simko L, Bohac M (2017). Carpal tunnel syndrome: symptoms, causes and treatment options. Literature reviev. Ortop Traumatol Rehabil.

